# Changing trends in gamma knife surgery to linear accelerator brain stereotactic radiotherapy in Japan: a survey based on the nationwide claims database

**DOI:** 10.1007/s11604-023-01511-1

**Published:** 2023-11-18

**Authors:** Yutaro Koide, Takahiro Aoyama, Hiroshi Tanaka, Yurika Shindo, Naoya Nagai, Tomoki Kitagawa, Hidetoshi Shimizu, Shingo Hashimoto, Hiroyuki Tachibana, Takeshi Kodaira

**Affiliations:** 1 Department of Radiation Oncology, Aichi Cancer Center Hospital, Kanokoden 1–1, Chikusa-ku, Nagoya, Aichi Japan; 2 Department of Radiation Oncology, Shiokawa Hospital Gamma Knife Center, Suzuka, Japan

**Keywords:** Stereotactic radiosurgery, Stereotactic radiotherapy, Brain metastases, Gamma knife, Linear accelerator

## Abstract

**Purpose:**

This study evaluated the trends in the platform for stereotactic radiotherapy to the brain (SRT), utilizing the open data of the National Database published by the Ministry of Health, Labour, and Welfare.

**Materials and methods:**

This study analyzed data from FY2014 to FY2021. The practices included in the study were gamma knife surgery (GKS) and SRT with a linear accelerator (LINAC). The total number of outpatient and inpatient cases in each SRT system was evaluated annually.

**Results:**

From April 2014 to March 2022, the study included 212,016 cases (102,691 GKS and 109,325 LINAC) of the registered 1,996,540 radiotherapy cases. In the first year, 13,117 (54.1%) cases were GKS, and 11,128 (45.9%) were LINAC; after that, GKS decreased, and LINAC increased, reaching the same rate in FY2017. Compared to the first year, the final year showed 11,702 GKS (− 1415 or − 10.8%) and 17,169 LINAC (+ 6041 or + 54.3%), with an increase of 4626 total SRT cases to 28,871 (+ 19.1%). The percentage of outpatient treatment also increased from 4.6 to 11.8% for GKS and from 44.7 to 57.9% for LINAC.

**Conclusion:**

The study found a gradual decrease in the selection of GKS, an increasing trend in the selection of LINAC, and an increase in the overall number of stereotactic irradiations. In particular, the proportion of outpatient treatment increased, indicating that more than half of LINAC was selected for outpatient treatment.

**Supplementary Information:**

The online version contains supplementary material available at 10.1007/s11604-023-01511-1.

## Introduction

With advances in cancer treatment, the prognosis of adult cancer patients improves, and the proportion of patients with brain metastases is expected to increase [1–6]. Stereotactic radiosurgery/radiotherapy (SRS/SRT) of the brain is a treatment modality expected to provide high local control while minimizing delayed brain injury or dysfunction. The SRS platform included a gamma knife (GK) and a linear accelerator (LINAC). GK was developed by Lars Leksell, a neurosurgeon at Karolinska University in Sweden, in 1968 [7–9]. It uses gamma rays emitted from approximately 200 sources (cobalt-60) without damaging the surrounding normal tissue and concentrates them in the lesion area. The first GK unit in Japan was introduced in 1990; after being covered by public insurance in 1996, it spread widely throughout the country [10]. Gamma Knife Surgery (GKS) is performed mainly by neurosurgeons belonging to the Japanese Leksell Gamma Knife Society (JLGK). The total number of GK units is currently 50, but a maximum of 55 units existed in the past (2011–2012 and 2016–2017) [11, 12]. Yamamoto and JLGK reported the total number of GKS cases in Japan up to 2013, with a cumulative total of approximately 200,000 treated cases (approximately 70% were malignant tumors) [11]. Although the annual number of GKS cases increased until 2006 (15,091 cases), when the government reduced reimbursements for GKS by 20% (from 63,000 to 50,000 points); it has decreased further since then.

LINAC-based brain SRT (LINAC-SRT) was introduced into clinical practice after GKS. Fixing the headframe used for GK is invasive. LINAC-SRT using a relocatable headframe was reported by Laing and Brada in 1994 and has rapidly spread worldwide [13, 14]. Under the Japanese universal health insurance system, all citizens and foreign residents must join the national health insurance system, and the government covers 70% of the healthcare costs. The patients paid for the remaining 30% (depending on age and other factors) [15]. Public insurance began to cover LINAC-SRT in FY1998, two years after GK. Although only 10% of the 649 LINAC in Japan at that time were capable of brain SRT, many replacements and the introduction of new LINAC have led to an increase in the use of brain SRT in Japan [16, 17]. According to the latest 2019 Japan Society for Radiation Oncology (JASTRO) survey, 697 valid responses were received from 960 LINAC facilities, with 308 (44%) patients undergoing brain SRT [18].

Real-world data on the national annual total number of SRS/SRT cases was difficult to obtain in Japan, especially LINAC-SRT, which in the past could only be estimated from the results of sampling databases from the JASTRO structural survey [16–33]. Computerizing health and medical information has recently progressed in Japan, and large and highly complete insurance claims databases have been established. Previously (from FY1996), one-month data from paper claim receipts extracted by stratified random two-stage sampling methods were published as the Statistics of Medical Care Activities in Public Health Insurance [34]. With the enforcement of the “Act on Assurance of Medical Care for Elderly People” of 2008, the National Health Insurance Claims and Specific Health Examination Database (NDB) was established as the complete enumeration database. Since FY2011, the NDB has been used secondarily for research purposes. As of July 2023, eight years of data from FY2014 to FY2021 are freely available as open NDB data [35–42]. The NDB has the advantage of being a huge, highly complete database that covers almost all insured medical treatment throughout Japan (99.9% since FY2011); because the data are collected from insurers, patients are tracked on an individual level, even if they switch to a different hospital.

This study aimed to investigate recent trends in selecting GKS and brain LINAC-SRT over time using the NDB open data. Secondary endpoints were changes in the number of treatments and facilities (GK and LINAC) over 30 years and disparities in GK facilities by prefecture, using data from the JASTRO and JLGK reports. The results of this study provide a comprehensive structure of the SRS/SRT in Japan as foundational data for future studies.

## Materials and methods

### Study design and data source

This retrospective, observational study used data from the National Insurance Claims Database. This study was approved by the Institutional Ethics Review Committee (2023-0-004). All studies were performed in accordance with relevant guidelines and regulations.

The primary endpoint was the total number of GKS and LINAC-SRTs in the country from FY2014 to FY2021, covered by the NDB open data. The secondary endpoint was defined as the change over time in the annual number of GKS and LINAC-SRT and the number of each unit from FY1990 to FY2013. The total number of GKS from FY1990 to FY2013 was reported by Yamamoto et al. [11]. The total LINAC-SRT was calculated using GKS data and JASTRO structural survey results [11, 16–33]. The total number of LINAC units in Japan for each year was compiled from the JASTRO structural survey [16–33].

The first structural survey of JASTRO was performed in 1990; since 1993, it has been surveyed every two years as an academic project (2009–2013). The latest version of this survey is the FY2019 edition [18]. Each LINAC hospital was required to cooperate with this survey for the accreditation of radiotherapy facilities by JASTRO, but JLGK did not penalize GK hospitals for not cooperating. The survey investigated the actual status of radiotherapy facilities by regional and prefectural epidemiology, including the number of facilities, types of radiotherapy equipment, facility size, number of treatment plans according to their complexity and annual patient load, number of staff, and annual cancer patients according to the patient load of radiation oncology institutions, radiotherapy other than external beam irradiation (e.g., stereotactic body radiotherapy, IMRT, and particle therapy), target diseases, and sites of treatment. Except in the 2003 edition, the Survey publishes only the total number of registered SRS/SRT cases without distinguishing between GK and LINAC, except for the 2003 edition [23]. In the latest survey (FY2019 edition) in September 2020, a formal request for a structural survey on the actual practice of radiotherapy during 2019 was made to 843 nationwide facilities that were assumed to have radiotherapy equipment by the Database Committee [18]. Some facilities suspended or stopped radiotherapy, and an estimated 842 facilities provided radiotherapy in 2019. Of these, 734 (87.2%) were included in the analysis. The survey results are presented only in figures and tables. Brain SRS/SRT using CyberKnife (CK) is considered LINAC-SRT as a medical procedure for insurance claims. The availability of CK in Japan is not significant, with only 31 units still in operation as of 2019 [18]. The following major historical insurance policies for GKS and LINAC-SRT in Japan are referred to when interpreting the results of secondary endpoints: FY1996 and FY1998, when GKS and LINAC-SRT were covered by public insurance, respectively. FY2000 was when the Ministry of Health and Welfare, the administrator of the NDB, was established because of the merger of ministries. FY2006 was when insurance GKS healthcare costs were reduced by 20%.

### Outcome measures and statistical analyses

In NDB analysis, the medical practice (classification code, practice code, insurance score) for GKS was defined as “Stereotactic radiotherapy with gamma knife” (M001-2, 180018910, 50,000). The following medical procedures that could be calculated in the case of hospitalization from FY2016 were also considered eligible: “basic fee for short-stay surgery 3” (A400, 190197910, 59,199–59,855). The medical practice for LINAC-SRT was defined as “Stereotactic radiotherapy with linear accelerator” (M001-3, 180019710, 63,000). Receipts were differentiated between outpatients and inpatients, and each was counted.

Before the introduction of the NDB open data for GKS, the annual number of GKS cases from FY1990 to FY2013 was extracted from Yamamoto et al. [11]. For LINAC-SRT, the number of cases was considered to be zero before insurance coverage (until FY1997), and the annual cases from FY1998 to FY2013 were calculated from the JASTRO structural survey reports and the JLGK reports, in which the total cases of GKS and LINAC-SRT were both available. The GKS report did not specify detailed data tables or tabulation methods; the figures were read from graphs and used in this study. The numbers when the annual number of LINAC-SRTs cannot be calculated are treated as missing values on the graphs but are described in the supplemental tables, referring to the JASTRO and JLGK reports.

We forecast the changing number of GKS and LINAC-SRT cases up to FY2030 based on the 8-year NDB data. The forecast assumes that demand will continue to change linearly through FY2030 within the degree of change per year from FY2014 to FY2021. The forecast shows a mean value of ± 2 SD. Although whether future change will continue to be linear, as per our assumption, is unclear, data as reliable as the NDB for estimating the rate of change are currently lacking; this study provided linear forecast results despite its limitations.

## Results

### Patients and primary endpoint

**Fig. 1:**
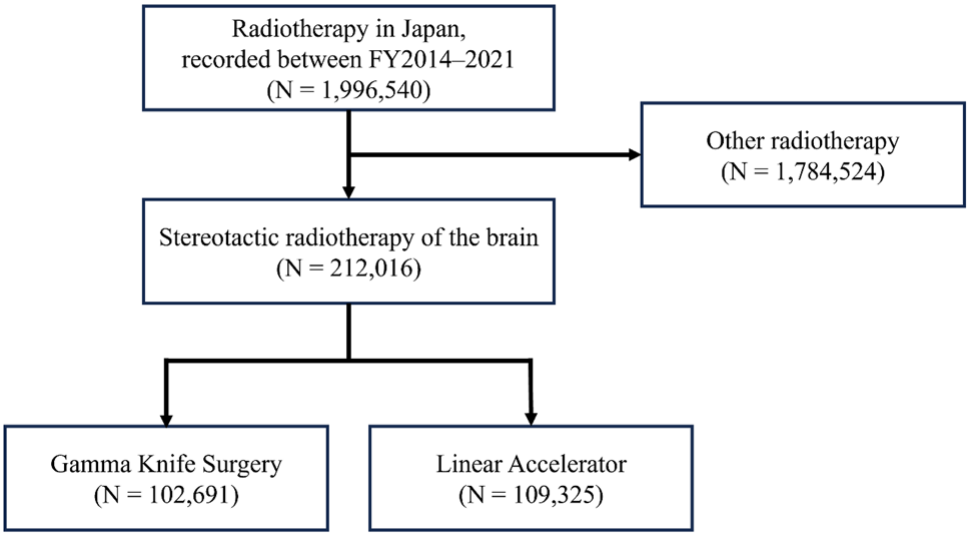
Flow diagram detailing the process of selecting the study cohort

**Fig. 2:**
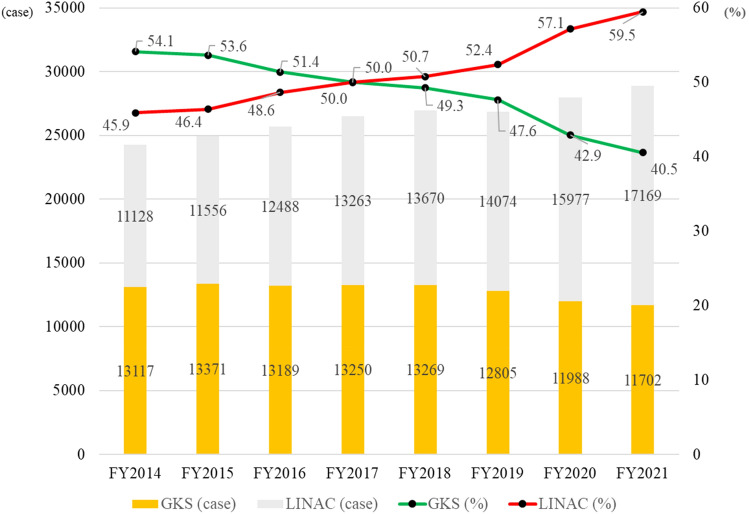
Gamma knife surgery and linear accelerator stereotactic radiotherapy, FY2014–2021. GKS: gamma knife surgery, LINAC: linear accelerator

**Fig. 3:**
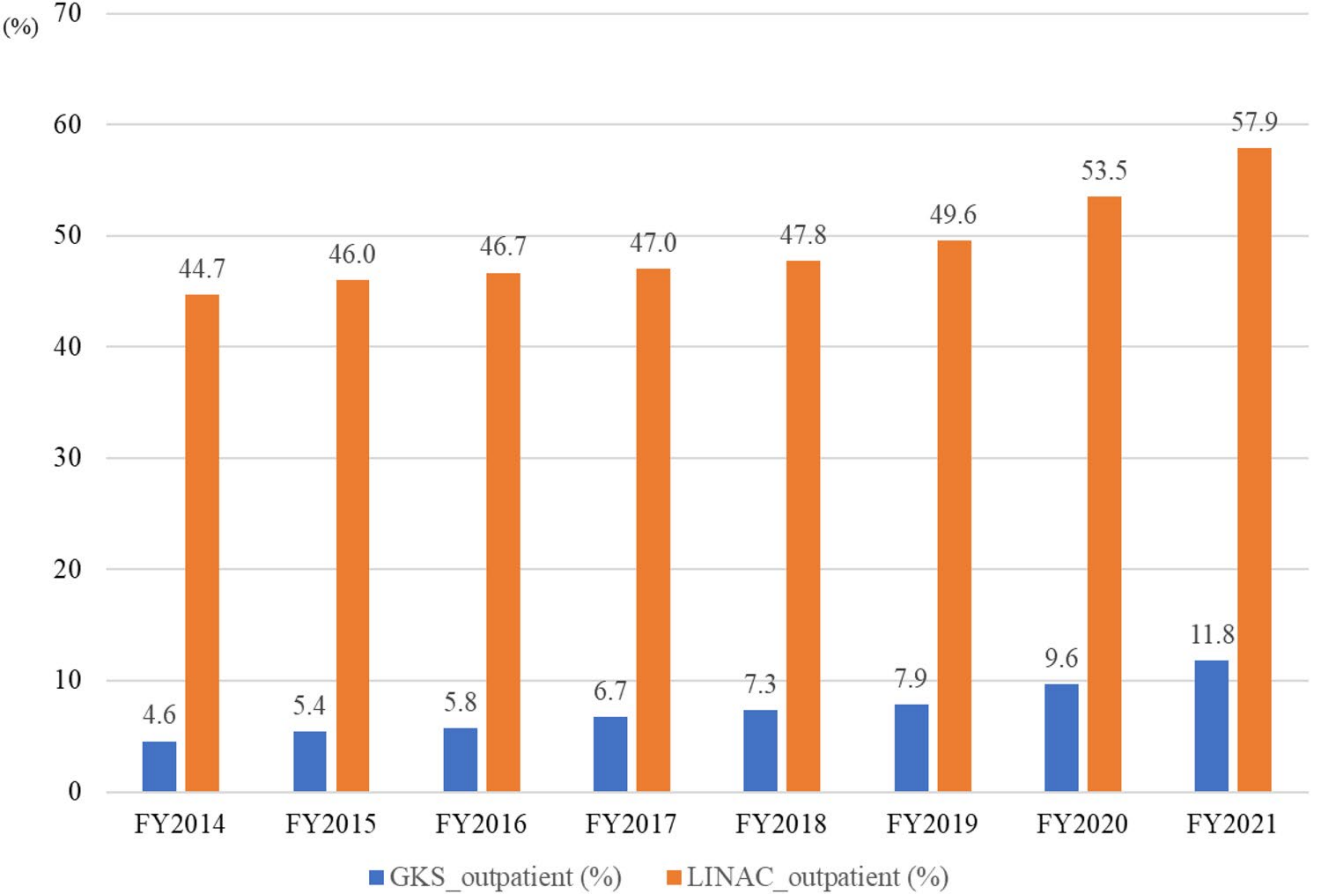
Percentage of outpatients in gamma knife surgery and linear accelerator stereotactic radiotherapy, FY2014–2021. GKS: Gamma Knife Surgery, LINAC: Linear Accelerator

Over the eight years from April 2014 to March 2022, 212,016 cases (102,691 GKS and 109,325 LINAC) of the total registered 1,996,540 radiotherapy cases were included (Fig. 1). Table 1 shows the total number of treatments and units for the GK and LINAC each year. In the first year, 13,117 (54.1%) cases were GKS, and 11,128 (45.9%) were LINAC. GKS decreased, while LINAC increased; they had the same rate in FY2017. Compared to the first year, the final year showed 11,702 GKS (− 1415 or − 10.8%) and 17,169 LINAC (+ 6,041 or + 54.3%) cases, with an increase from 4626 total SRS/SRT cases to 28,871 (+ 19.1%, Fig. 2). The percentage of outpatient treatment also increased from 4.6 to 11.8% for GKS and from 44.7 to 57.9% for LINAC (Fig. 3).

**Table 1:** Gamma knife surgery and linear accelerator stereotactic radiotherapy, FY2014–2021

Year (FY)	GKS (outpatient %)	GK units	LINAC SRT (outpatient %)	LINAC units (% of SRT available)
2014	13,117 (4.6)	54	11,128 (44.7)	NA
2015	13,371 (5.4)	54	11,556 (46.0)	223 (32.1)
2016	13,189 (5.8)	55	12,488 (46.7)	NA
2017	13,250 (6.7)	55	13,263 (47.0)	267 (39.1)
2018	13,269 (7.3)	54	13,670 (47.8)	NA
2019	12,805 (7.9)	54	14,074 (49.6)	308 (44.1)
2020	11,988 (9.6)	54	15,977 (53.5)	NA
2021	11,702 (11.8)	52	17,169 (57.9)	NA
2022	NA	50	NA	NA
2023	NA	50	NA	NA

*GKS*, gamma knife surgery; *GK*, gamma knife; *LINAC*, linear accelerator; *SRT*, stereotactic radiotherapy

### Secondary endpoint

**Fig. 4:**
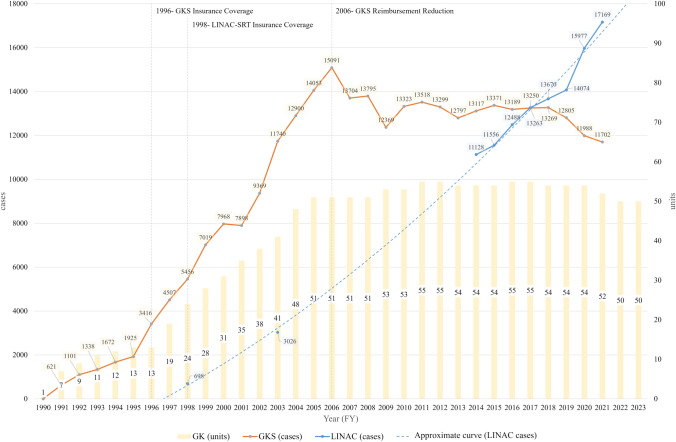
Changes in gamma knife surgery, units, and linear accelerator stereotactic radiotherapy, FY1990–2021. GKS: gamma knife surgery, LINAC: linear accelerator

From FY1990 to FY2013, the total number of GKS was 198,879, rapidly growing from FY1996 with an increase of 1000–1500 GKS and 3–7 GK units per year (except FY2000–2001), until peaking in FY2006 (Table S1). After FY2006, the use of GKS decreased; GK units increased to a maximum of 55 units in FY2016. Since then, five units have closed; thus, the total number of units has decreased to 50 (Fig. 4).

The annual number of LINAC-SRTs was mentioned clearly only in the 2003 edition of the JASTRO Structural Survey Report: In the 40 GK units (100%), and 155 LINAC were capable of SRT out of 695 valid responses (87.5%), 9584 GKS and 3026 LINAC-SRTs were inputted [23]. A total of 49 LINAC did not answer. Although not specified in the JASTRO Structural Survey report, the total number of cases in 1998 was estimated from these two reports [20]. The JASTRO report registered 6154 SRS/SRT (the sum of GKS and LINAC-SRT) and 18 GK units (100% in that year). Furthermore, 5456 GKS were registered in the GKS database, and the number of LINAC-SRT was calculated to be 698. Unfortunately, the JASTRO structural survey reports for the other eight years (2001, 2005, 2007, 2009, 2010, 2011, 2012, and 2013) contained the sum of the GKS and LINAC-SRT. However, LINAC-SRT could not be calculated as in 1998 [22, 24–31] because the GKS and LINAC-SRT were not fully registered, and the registered figures did not match those of the JLGK report. Table S1 lists the total numbers of SRS/SRT cases and units (GK and LINAC).

Figure S1 shows the changes in the total number of LINAC capable of performing brain SRT. In the JASTRO structural survey in FY1995, only 37 (7.4%) of the 499 valid responses were from 608 LINAC [16]. The total number of SRT-capable LINAC increased annually from 7.4 to 44% among the valid responses between FY1995 and FY2019.

Of the 47 prefectures in Japan, 10 had two or more GK facilities, and 27 had one GK. The other 10 prefectures have no GK facilities, including four that previously had facilities but are yet to be established (Figure S2). According to the public information of GK facilities and the year of the medical license registry for each GK surgeon, a median of 1 (range: 1–4) GK surgeons (neurosurgeons) were involved in each institution, with a mean age of 57 years (standard deviation 9.8, median 57 years, IQR: 51–62 years); 17.6% of the total population was under 50 years, 43.2% were 50–59 years, and 29.7% were 60–69 years [12].

## Discussion

This study is the first to evaluate the annual trends in the total number of GKS and LINAC-SRTs across Japan using the 8 years’ NDB open data [35–42], a report on the total number of GKS for 23 years by JLGK [11], and JASTRO structural survey reports published every two years for 30 years [16–33]. Because the NDB data were available, we now have access to its huge, neutral, and complete enumeration data concerning the number of LINAC-SRTs and GKS. These results strongly indicate that the previously growing GKS is now in decline, and LINAC-SRT has already emerged as the most selected treatment platform, replacing it.

Several factors may explain the shift from GK to LINAC as the brain SRS/SRT modality. Recent improvements in LINAC-SRT technology, including micro multileaf collimators (MLC) and single isocenter multi-target non-coplanar SRS/SRT systems, may have been the reason for the rise in LINAC-SRT [44–49]. Micro-MLCs allow precise radiation beam control, minimizing exposure to surrounding normal tissues while concentrating high doses on tumors [43–45]. Single-isocenter systems enable efficient simultaneous treatment of multiple tumors, significantly reducing treatment time without increasing the risk of local failure [46–48]. However, a major reason for the decline in GKS cases was the effect of the 20% reduction in GKS reimbursement fees (from 63,000 to 50,000 dollars) in 2006. Furthermore, since 2006, GK facilities have been unevenly distributed geographically, with the number of prefectures without a single GK facility increasing to 10 (21%); the aging of GK surgeons and the lack of a successful generational transition may also have contributed to the subsequent decline (Figure S2).

Another reason for choosing LINAC-SRT over GKS is access to facilities. LINACs are currently deployed in Japan nearly 20 times more frequently than GK, making treatment more readily available to patients. In addition, LINACs are located in cancer centers or academic centers that are the primary providers of oncology care, and patients do not need to be transferred to other facilities when newly diagnosed with brain metastases, expecting them to shift from brain SRS/SRT to resume or start systemic treatment quickly. According to the Ministry of Health, Labour, and Welfare, as of April 1, 2023, Japan has 51 core hospitals for cancer treatment in prefectural units and 357 in regional units that must provide radiation therapy, including IMRT, as a designation requirement [49]. Among these prefectural or regional core hospitals, only 11 had GK units; the remaining 397 had one or more LINAC. In contrast, GK plays an essential role in brain SRS/SRT in the USA, as recently as 2019, with 113 (26%) GK in 428 SRS systems [50]. A significant difference from Japan was that GK was the most implemented SRS system in academic centers (38%) in the USA.

Another contributing factor may be that while more than half of LINAC are now chosen for outpatient treatment, only 10% of GKS are treated on an outpatient basis. Because of the invasive headframe fixation of the GKS, all patients required inpatient treatment until 2015. Although faster treatment and noninvasive fixation with a soft-shell mask have been available since Gamma Knife Perfection™ and Icon™ were introduced in Japan in December 2008 and October 2016, respectively, the ratio of outpatient treatment remains limited compared to LINAC [11, 12].

The number of brain SRS/SRTs has continued to increase over the past 30 years; therefore, preparing them in advance is necessary for operating appropriate platforms to meet future demands. If the demand continues to increase linearly at a rate within the range observed over the past 8 years, the annual demand for brain SRS/SRT is forecasted to be 35,500 cases in 2030: LINAC-SRT at 27,000 cases and GKS at 8500 cases (Figure S3). With > 50% of LINACs in Japan still not SRT-available, there is an urgent need to promote the replacement of compatible models in prefectures where the GK is expected to close. If GKs are shut down further, the brain SRS/SRT supply may suddenly become critical locally; frequent updating and sharing of regional or prefectural situations are necessary to balance the SRS/SRT supply.

This study had several limitations. First, it used multiple data sources with different data reliabilities. Although the NDB is the country’s largest and most complete data source, covering nearly 99% of all insurance practices, only recent data from the last eight years are available. Compared to the NDB, the JASTRO Structural Survey and the JLGK Report, which are self-administered surveys, showed discrepancies, but this study could not explain them. Second, this study observed only treatment structure data, such as annual treatment numbers and facilities, and not patient outcomes (e.g., survival and tumor control) and did not compare the clinical efficacy or safety of GKS and LINAC-SRT. In addition, this study observed that GKS and LINAC-SRT were the only treatments observed; whole-brain radiotherapy (WBRT) was not observed, and WBRT could not be extracted as an independent insurance practice from the insurance claims database, unlike SRS/SRT.

In conclusion, this study identified a trend over the last 8 years in the selection of GKS and LINAC-SRT in Japan, with a 20% increase in overall brain SRS/SRT, a 10% decrease in GKS, and a > 50% increase in LINAC-SRT. The proportion of outpatients also increased, accounting for 10% of GKS patients and > 50% of LINAC-SRT patients. The results of this survey should be utilized to balance platforms appropriately to meet the national demand for SRS/SRT.

### Supplementary Information

Below is the link to the electronic supplementary material.

Supplementary file1 (DOCX 670 KB)

## Data Availability

Research data are stored in an institutional repository and anonymized numerical data will be shared upon request to the corresponding author.
