# Supplementary material for: Changing trends in gamma knife surgery to linear accelerator brain stereotactic radiotherapy in Japan: a survey based on the nationwide claims database
Source: Jpn J Radiol. 2023 Nov 18;42(4):415–23. doi: 10.1007/s11604-023-01511-1 (PMC10980651; doi:10.1007/s11604-023-01511-1)

**Supplementary files**

**Table S1**. **Structural survey results of the Japanese Society for Radiation Oncology and a report of the Japanese Leksell Gamma Knife Society, FY1990–2019**

|  | **JASTRO structure survey** | | | | | | **JLGK** |
| --- | --- | --- | --- | --- | --- | --- | --- |
| Year | LINAC  (SRT) | LINAC  (No SRT) | LINAC  (Not answered) | LINAC  (Overall) | GK units  (enrolled/overall) | SRS/SRT cases  (GKS+LINAC) | GKS cases |
| 1990 | 0 | 311 | 156 | 467 | NA/1 | NA | 0 |
| 1991 | NA | NA | NA | NA | NA/7 | NA | 621 |
| 1992 | NA | NA | NA | NA | NA/9 | NA | 1101 |
| 1993 | 0 | 508 | 33 | 541 | NA/11 | NA | 1338 |
| 1994 | NA | NA | NA | NA | NA/12 | NA | 1672 |
| 1995 | 37 | 462 | 109 | 608 | 5/13 (38%) | NA | 1925 |
| 1996 *^1^ | NA | NA | NA | NA | NA/13 | NA | 3416 |
| 1997 | 48 | 502 | 99 | 649 | 18/19 (95%) | NA | 4507 |
| 1998 *^2^ | NA | NA | NA | NA | NA/24 | NA | 5456 |
| 1999 | 84 | 524 | 66 | 674 | 28/28 (100%) | 6154 | 7019 |
| 2000 | NA | NA | NA | NA | NA/31 | NA | 7968 |
| 2001 | 210 | 360 | 125 | 695 | 33/35 (94%) | 7737 | 7898 |
| 2002 | NA | NA | NA | NA | NA/38 | NA | 9369 |
| 2003 | 155 | 540 | 49 | 744 | 40/41 (98%) | 12610 (9584/3026) | 11740 |
| 2004 | NA | NA | NA | NA | NA/48 | NA | 12900 |
| 2005 | 149 | 515 | 101 | 765 | 48/51 (94%) | 11122 | 14053 |
| 2006 | NA | NA | NA | NA | NA/51 | NA | 15091 |
| 2007 | 140 | 535 | 132 | 807 | 46/51 (90%) | 12554 | 13704 |
| 2008 | NA | NA | NA | NA | NA/51 | NA | 13795 |
| 2009 | 156 | 498 | 162 | 816 | 46/53 (87%) | 13855 | 12369 |
| 2010 | 165 | 494 | 170 | 829 | 46/53 (87%) | 13800 | 13323 |
| 2011 | 168 | 480 | 188 | 836 | 46/55 (84%) | 13768 | 13518 |
| 2012 | 189 | 476 | 199 | 864 | 44/55 (80%) | 14450 | 13299 |
| 2013 | 206 | 466 | 208 | 880 | 45/54 (83%) | 15828 | 12797 |
| 2014 | NA | NA | NA | NA | NA/54 | NA | 13117 |
| 2015 | 223 | 471 | 242 | 936 | 43/54 (80%) | 14910 | 13371 |
| 2016 | NA | NA | NA | NA | NA/54 | NA | 13189 |
| 2017 | 267 | 415 | 266 | 948 | 35/54 (65%) | 18484 | 13250 |
| 2018 | NA | NA | NA | NA | NA/54 | NA | 13269 |
| 2019 | 308 | 389 | 263 | 960 | 37/54 (69%) | 19315 | 12805 |

JASTRO, Japanese Society for Radiation Oncology; JLGK, Japanese Leksell Gamma Knife Society; GKS, gamma knife surgery; Gamma Knife LINAC, linear accelerator; SRS, stereotactic radiosurgery; SRT, stereotactic radiotherapy

*^1^ From FY1996, GKS was covered by public insurance.

*^2^ From FY1998, LINAC-based SRT was covered by public insurance.

**Figure S1. Changes in Linear Accelerator for Stereotactic Radiotherapy, FY1990–2019**

LINAC: Linear Accelerator


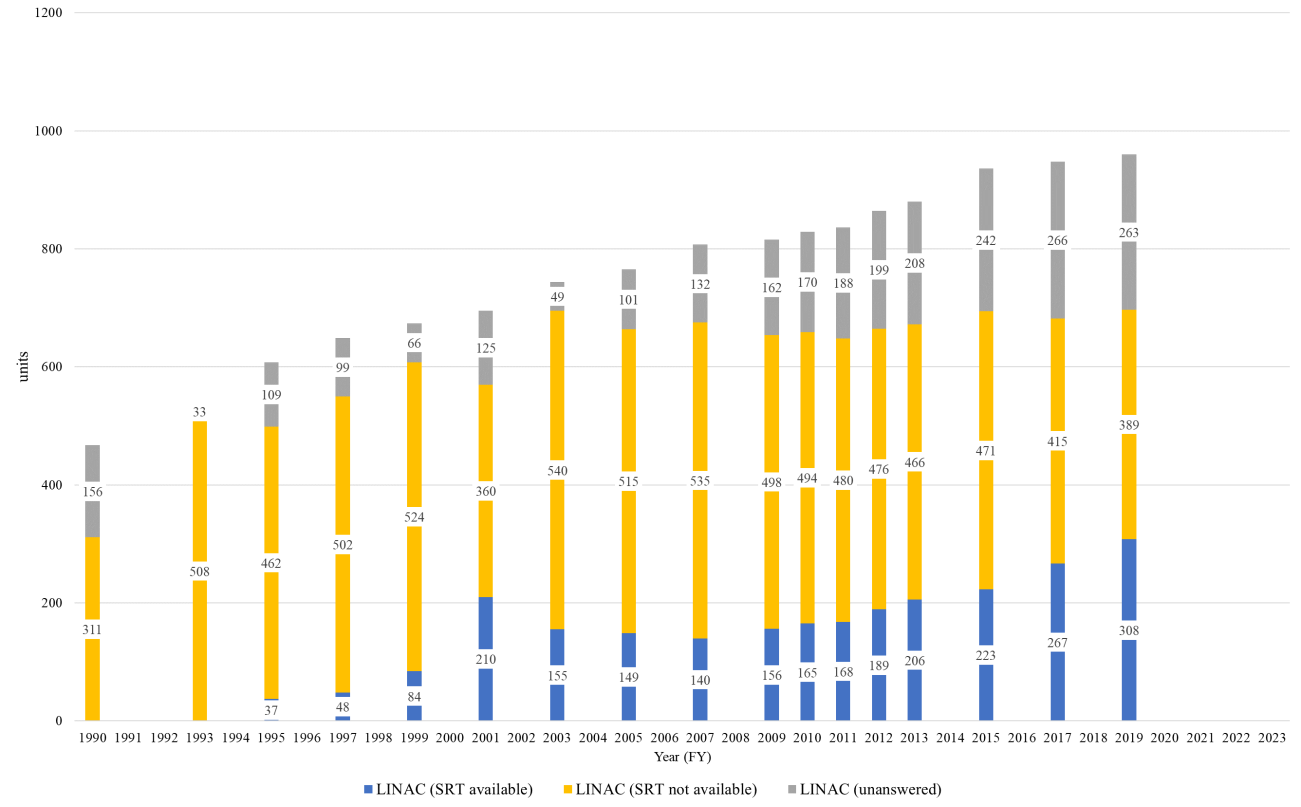


**Figure S2. Distribution of Gamma Knife in Japan, FY2023**


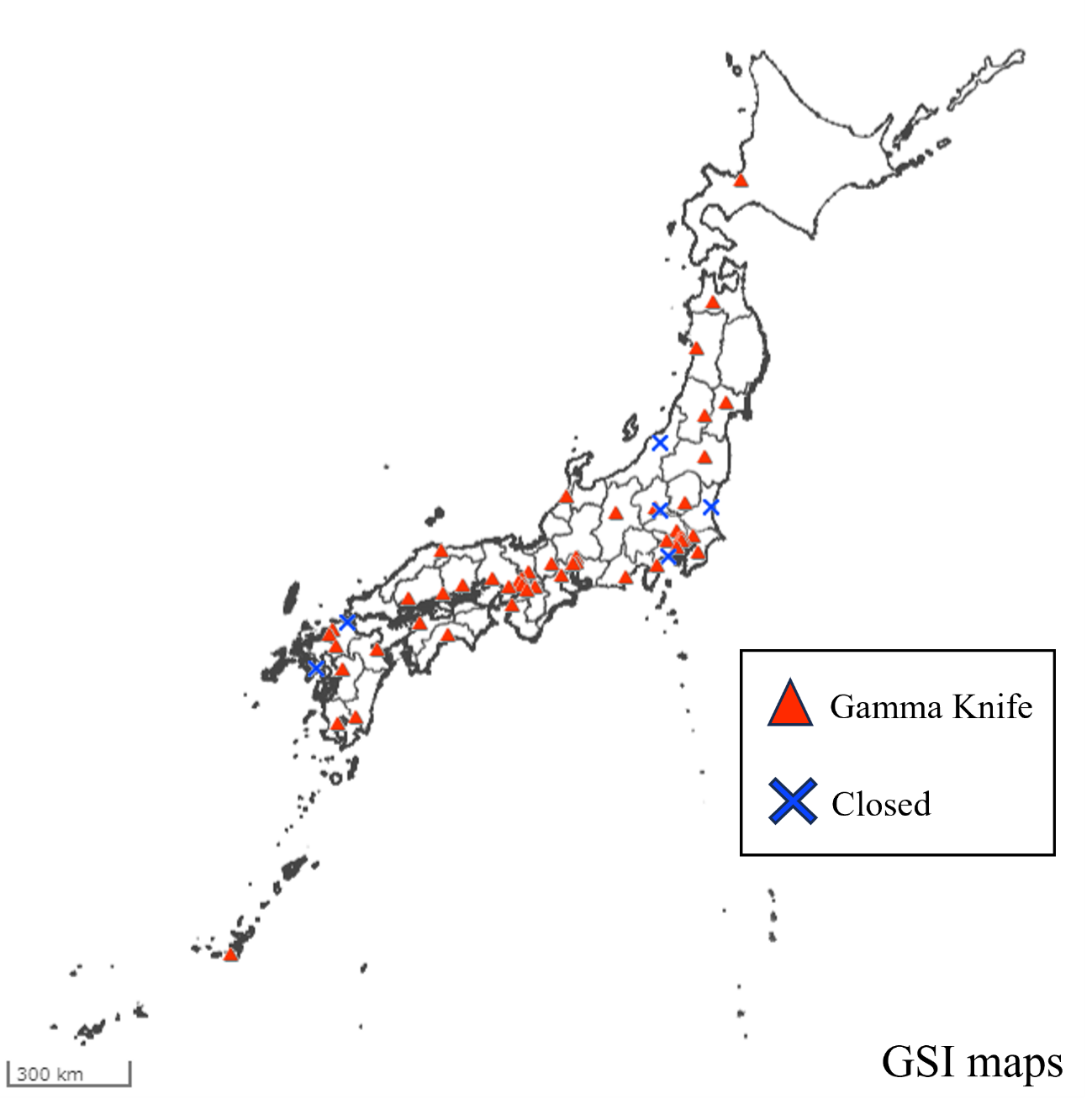


**Figure S3**. Forecast data of Gamma Knife Surgery and Linear Accelerator Stereotactic Radiotherapy in Japan, FY2022–2030


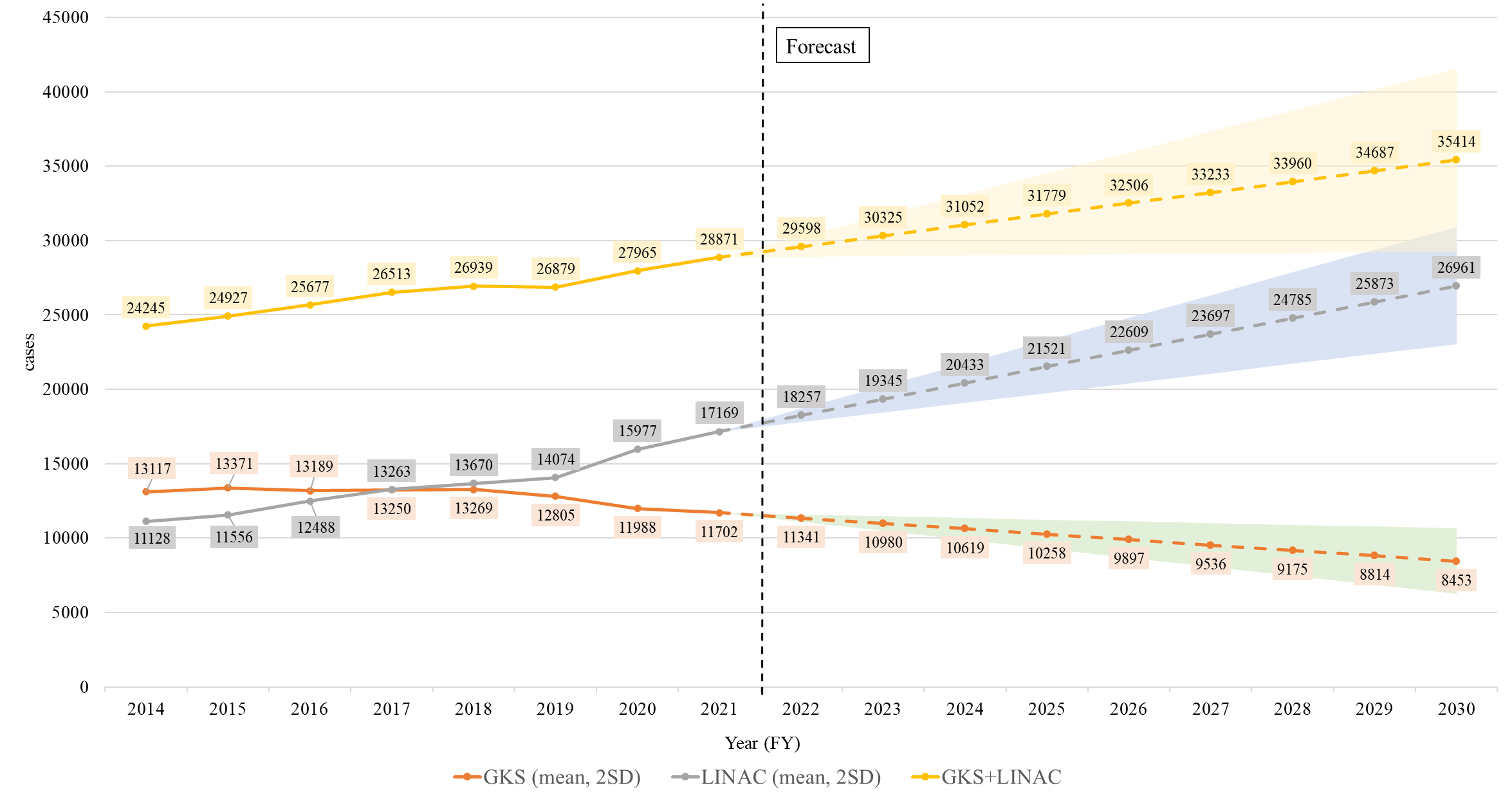

Supplement: Supplementary file 1 — Supplementary file1 (DOCX 670 KB) [file 11604_2023_1511_MOESM1_ESM.docx]
